# Acute Infections, Antibiotic Use and Antibiotic-Related Knowledge Among People Living with HIV: A Cross-Sectional Questionnaire-Based Study

**DOI:** 10.3390/antibiotics15100979

**Published:** 2026-10-03

**Authors:** Adrian Gabriel Marinescu, Cătălin Tilișcan, Aida-Isabela Adamescu, Daniela Pițigoi, Sebastian Ciobanu, Laurențiu Mihăița Stratan, Dalila Toma, Carmen Cristina Vasile, Victoria Aramă, Ștefan Sorin Aramă

**Affiliations:** 1 Faculty of Medicine, Department II, Discipline of Infectious Diseases II, Carol Davila University of Medicine and Pharmacy, 37 Dionisie Lupu Street, 020021 Bucharest, Romaniamihaita.stratan@drd.umfcd.ro (L.M.S.);; 2 Department of Infectious Diseases, National Institute of Infectious Diseases “Prof. Dr. Matei Bals”, 020021 Bucharest, Romaniasorin.arama@umfcd.ro (Ș.S.A.); 3 Faculty of Dental Medicine, Department II, Discipline of Pathophysiology and Immunology, Carol Davila University of Medicine and Pharmacy, 37 Dionisie Lupu Street, 020021 Bucharest, Romania; 4 Department of Radiology and Medical Imaging, Emergency University Hospital, 050098 Bucharest, Romania

**Keywords:** HIV, people with HIV, antibiotic use, antimicrobial stewardship, antibiotic knowledge, health literacy

## Abstract

**Background:** Acute infections and antibiotic use remain clinically relevant among people living with HIV (PLWH). This exploratory study assessed self-reported acute infections, usual healthcare contact, antibiotic use, and antibiotic-related knowledge among PLWH. **Methods:** We conducted a single-center cross-sectional questionnaire-based study among 209 PLWH in Bucharest, Romania (October 2025–March 2026). The questionnaire assessed self-reported acute infections and hospitalizations during the preceding 12 months, usual healthcare contact, antibiotic use, and six knowledge items. Frequent self-reported antibiotic use was defined as use on at least three occasions during the preceding 12 months. **Results:** At least one self-reported acute infection was reported by 45.5% of participants; 9.6% reported an infection-related hospitalization. The family physician was the most frequently reported usual healthcare contact (55.0%), followed by the infectious diseases physician (31.6%). Among 207 participants with evaluable antibiotic-use data, 22 (10.6%) met the frequent-use definition. In exploratory analyses, knowledge scores were higher among participants with greater educational attainment and those living in urban areas, but showed no clear evidence of an association with frequent self-reported antibiotic use (unadjusted OR per 1-point increase, 1.16; 95% CI 0.88–1.54; *p* = 0.290). In an exploratory adjusted analysis, self-reported acute infection and suboptimal physician-estimated antiretroviral therapy (ART) adherence were associated with frequent self-reported antibiotic use. **Conclusions:** Frequent self-reported antibiotic use was associated with reported acute infections and suboptimal physician-estimated ART adherence, whereas there was no clear evidence of an association with antibiotic-related knowledge. This pattern suggests that infectious morbidity and care-related factors may be important correlates of repeated antibiotic use in PLWH and highlights the potential value of coordinated stewardship across HIV and primary care.

## 1. Introduction

The widespread use of effective antiretroviral therapy (ART) has fundamentally changed the prognosis of human immunodeficiency virus (HIV) infection, transforming it from a frequently fatal disease into a manageable chronic condition. Among adults receiving long-term ART in Europe and North America, life expectancy has approached that of the general population, particularly in individuals with sustained viral suppression and preserved CD4 cell counts [1]. Nevertheless, important gaps in comorbidity-free survival remain [2], and persistent immune activation may contribute to residual non-AIDS morbidity despite effective treatment [3,4].

As survival has improved, the clinical spectrum of HIV care has expanded beyond opportunistic infections to include common community-acquired infections and non-AIDS-related infectious morbidity. Respiratory tract infections and community-acquired pneumonia remain clinically relevant among people living with HIV (PLWH), although their incidence and outcomes are influenced by immune status, viral suppression, age, comorbidities, and vaccination [5,6,7]. Severe bacterial non-AIDS infections, particularly pneumonia, bloodstream infections, and urinary tract infections, remain important causes of hospital admission [8]. Acute viral infections may also contribute to longer-term morbidity [9].

Antibiotics remain essential for the treatment of bacterial infections, but unnecessary or inappropriate antimicrobial exposure is a major driver of antimicrobial resistance (AMR). Globally, bacterial AMR was estimated to be directly responsible for 1.27 million deaths and associated with 4.95 million deaths in 2019 [10]. PLWH may be particularly vulnerable to resistant bacterial infections because of repeated healthcare contact, previous antimicrobial exposure, prophylactic treatments, and the cumulative effects of immune dysfunction and comorbidity. A systematic review and meta-analysis found higher odds of colonization or infection with several resistant bacterial pathogens among PLWH compared with people without HIV [11]. Despite these concerns, evidence describing outpatient antibiotic use among PLWH remains limited. Studies focused on acute respiratory illnesses have reported substantial inappropriate prescribing in both people with and without HIV and have suggested that prescribing quality may depend more on the treating clinician and care setting than on HIV status alone [12]. Other cohort data indicate that the frequency of acute respiratory infection encounters is higher among PLWH with unsuppressed viral load or lower CD4 cell counts, while inappropriate antibiotic use remains common across HIV subgroups [13]. These findings support evaluating antibiotic use together with infectious morbidity, HIV-related clinical factors, and healthcare-seeking pathways.

Appropriate antibiotic use is also influenced by patients’ knowledge, expectations, and access to reliable medical information. The World Health Organization AWaRe framework emphasizes evidence-based antibiotic selection and stewardship across outpatient and hospital settings [14]. In Romania, population-based studies have documented persistent misconceptions regarding antibiotic indications, premature treatment discontinuation, use of leftover antibiotics, and differences in knowledge according to education and place of residence [15,16]. Similar knowledge gaps have been reported across the WHO European Region, with physicians remaining the most trusted source of information [17]. Romanian family physicians have likewise identified insufficient patient education and public awareness as important drivers of antibiotic consumption and inappropriate expectations [18].

Romania has a long-standing and distinctive HIV epidemic, with 18,768 people living with HIV reported in 2024 and high ART coverage, while recent epidemiological trends increasingly resemble those observed elsewhere in Europe [19]. Few studies have jointly examined the frequency of acute infections, healthcare-seeking behavior, antibiotic use, and antibiotic-related knowledge among PLWH, particularly in Central and Eastern Europe.

The present study therefore aimed to characterize self-reported acute infections and infection-related hospitalizations, describe usual healthcare-contact patterns and antibiotic-use patterns, assess antibiotic-related knowledge and misconceptions, and explore factors associated with frequent self-reported antibiotic use among adult PLWH receiving routine care at a specialized infectious diseases center in Romania.

## 2. Results

### 2.1. Study Population Characteristics

During the study period, 227 eligible PLWH were approached; 18 declined participation and 209 completed the questionnaire and were included in the final analysis (participation rate 92.1%). The cohort was predominantly male, with 155 men (74.2%) and 54 women (25.8%). The mean age of participants was 42.2 ± 12.1 years, with a median age of 40 years (range: 20–77 years). Most participants originated from urban areas (77.0%, n = 161), while 23.0% (n = 48) were from rural areas.

Regarding educational status, high school education was the most frequently reported level (45.9%, n = 96), followed by university-level education (30.1%, n = 63). Lower educational levels were less frequent, including secondary school education in 15.8% (n = 33), primary school in 4.3% (n = 9), and no formal education in 3.3% (n = 7) of participants (data unavailable for 1 participant). With respect to occupational status, 42.1% (n = 88) of participants were employed, while 38.3% (n = 80) were retired. Unemployment was reported by 7.2% (n = 15), while 10.0% (n = 21) reported no occupation; students represented 1.9% (n = 4) of the cohort (data unavailable for 1 participant). Long-standing HIV infection was common in the study population: duration of HIV diagnosis exceeded 10 years in 45.5% (n = 95) of cases, while 26.8% (n = 56) had been diagnosed for 1–5 years, 18.7% (n = 39) for 6–10 years, and 9.1% (n = 19) for less than one year. Similarly, prolonged ART exposure was common in the cohort, with 43.1% (n = 90) of participants reporting more than 10 years since ART initiation. Participant characteristics are summarized in Table 1.

Among the 190 participants with available HIV-1 RNA data, an undetectable result (“target not detected”) was recorded in 140/190 (73.7%), while 39/190 (20.5%) had detectable HIV-1 RNA < 1000 copies/mL and 11/190 (5.8%) had HIV-1 RNA > 1000 copies/mL; HIV-1 RNA data were unavailable for 19/209 (9.1%). Among the 194 participants with available CD4 data, counts > 500 cells/mm^3^ were present in 129/194 (66.5%), 351–500 cells/mm^3^ in 33/194 (17.0%), 200–350 cells/mm^3^ in 18/194 (9.3%), and <200 cells/mm^3^ in 14/194 (7.2%); CD4 data were unavailable for 15/209 (7.2%).

Among the 193 participants with evaluable physician-estimated ART adherence, adherence was ≥95% in 173/193 (89.6%), 80–94% in 15/193 (7.8%), and <80% in 5/193 (2.6%); adherence data were unavailable or could not be reliably estimated for 16/209 (7.7%). Among the 191 participants with available treatment-interruption data, interruptions during the preceding 12 months were reported for 13/191 (6.8%), while 178/191 (93.2%) had no documented interruption; data were unavailable for 18/209 (8.6%). Among the 163 participants with available opportunistic-infection history, a history of opportunistic infection was recorded for 11/163 (6.7%) and absent for 152/163 (93.3%); data were unavailable for 46/209 (22.0%). These unavailable historical data were retained as missing rather than inferred to indicate absence.

Coinfection/comorbidity information was available for 193 participants; 94/193 (48.7%) had at least one recorded condition. The most frequent predefined categories were cardiovascular disease (20/193, 10.4%), hepatitis B virus infection (19/193, 9.8%), hepatitis C virus infection (14/193, 7.3%), diabetes (12/193, 6.2%), obesity (11/193, 5.7%), tuberculosis (10/193, 5.2%), chronic obstructive pulmonary disease (8/193, 4.1%), and neoplasia (3/193, 1.6%); categories were not mutually exclusive. Current ART regimen data were also available for 193 participants. The most frequent regimens were bictegravir/emtricitabine/tenofovir alafenamide (72/193, 37.3%), dolutegravir/lamivudine (41/193, 21.2%), doravirine/lamivudine/tenofovir disoproxil (36/193, 18.7%), and dolutegravir/abacavir/lamivudine (17/193, 8.8%); 27/193 (14.0%) had other or unspecified regimens. Additional descriptive clinical information is provided in Supplementary File S3.

### 2.2. Acute Infectious Diseases

During the preceding 12 months, 95/209 participants (45.5%, 95% CI 38.8–52.2%) reported at least one category of acute infection. Among the broad infection categories, respiratory infections were most frequently reported, affecting 60/209 (28.7%, 95% CI 23.0–35.2%), followed by digestive infections in 21/209 (10.0%), other acute infections in 21/209 (10.0%), and urinary infections in 20/209 (9.6%). Overall, 24/209 (11.5%) reported infections involving at least two different categories. As multiple responses were permitted, these categories were not mutually exclusive. In a separate questionnaire item addressing predefined specific diagnoses, urinary tract infection was reported by 17/209 (8.1%), while influenza and acute gastroenteritis were each reported by 16/209 (7.7%). Acute sinusitis was reported by 15/209 (7.2%), acute pneumonia by 11/209 (5.3%), COVID-19 by 8/209 (3.8%), and acute otitis by 7/209 (3.3%).

Hospitalization for an acute infection during the preceding 12 months was reported by 20/209 participants (9.6%, 95% CI 6.3–14.3%). Most participants reported no hospitalization (189/209, 90.4%); 15/209 (7.2%) reported one hospitalization, 3/209 (1.4%) reported two to three, and 2/209 (1.0%) reported at least four. There was no statistical evidence of a difference in hospitalization between participants with CD4 counts ≤ 350 cells/mm^3^ and those with CD4 counts > 350 cells/mm^3^ (3/32 [9.4%] vs. 16/162 [9.9%]; OR 0.94, 95% CI 0.26–3.45; Fisher’s exact *p* = 1.00), but the small number of events resulted in substantial imprecision. Likewise, no clear evidence of a difference in self-reported acute infection occurrence was detected between participants with detectable and undetectable HIV-1 RNA (52.0% vs. 44.3%, respectively; *p* = 0.348).

### 2.3. Usual Healthcare Contact for Acute Infections

All 209 participants were asked whom they usually contact when experiencing an acute infection, irrespective of whether they had reported an acute infection during the preceding 12 months. The family physician was the most frequently selected usual healthcare contact (55.0%, n = 115), followed by the infectious diseases physician (31.6%, n = 66). Emergency departments were selected by 6.7% (n = 14), another medical specialist by 2.9% (n = 6), self-treatment by 2.4% (n = 5), and a pharmacist by 1.4% (n = 3) of participants (Table 2).

The distribution of usual healthcare contact differed according to educational level (*p* = 0.0006). Participants with university-level or high-school education more frequently selected their family physician (58.7% and 59.4%, respectively), whereas participants with lower educational levels more frequently selected their infectious diseases specialist. The infectious diseases physician was selected by 55.6% of participants with primary school education and by 42.9% of those without formal education, compared with 25.4% among participants with university-level education.

### 2.4. Antibiotic Use Patterns

During the preceding 12 months, 99/209 participants (47.4%) reported no antibiotic use, 86/209 (41.1%) reported one to two occasions, 17/209 (8.1%) reported three to five occasions, and 5/209 (2.4%) reported more than five occasions. Two participants (1.0%) could not recall their antibiotic use. Among the 207 participants with evaluable responses, frequent self-reported antibiotic use (≥3 occasions) was reported by 22/207 (10.6%, 95% CI 7.1–15.6%).

Frequent self-reported antibiotic use was more common among participants with CD4 counts ≤ 350 cells/mm^3^ than among those with higher CD4 counts (7/32 [21.9%] vs. 15/161 [9.3%]; OR 2.73, 95% CI 1.01–7.35); however, Fisher’s exact testing provided no clear statistical evidence of a difference in this sample (*p* = 0.062), and the comparison was imprecise. More than half of participants with CD4 counts > 500 cells/mm^3^ (50.4%) reported no antibiotic use during the preceding 12 months.

Frequent self-reported antibiotic use was reported by 6/19 (31.6%) participants with physician-estimated ART adherence < 95% and evaluable antibiotic-use data, compared with 16/173 (9.2%) of those with adherence ≥ 95% (Fisher’s exact *p* = 0.011). Within the <95% group, 6/14 (42.9%) participants with adherence 80–94% met the frequent-use definition; one of the 15 participants in this adherence category had a non-evaluable antibiotic-use response. None of the 5 participants with adherence < 80% met the frequent-use definition. Among the 108 participants who reported antibiotic use, the single selected source of prescription or recommendation was most often the family physician (41/108, 38.0%), followed by another medical specialist (32/108, 29.6%) and the infectious diseases physician (25/108, 23.1%). Self-medication was selected by 6/108 (5.6%) and pharmacist recommendation by 4/108 (3.7%). No clear evidence of an association was observed between sex or area of residence and frequent self-reported antibiotic use.

### 2.5. Antibiotic-Related Knowledge and Item Responses

Responses to the antibiotic-related items varied considerably across the study population. Overall, 51.2% (n = 107) correctly recognized that antibiotics are effective only against bacterial infections, while 15.3% (n = 32) considered this statement false and 33.5% (n = 70) answered “do not know”. Only 42.1% (n = 88) correctly rejected the use of antibiotics for viral respiratory infections; 27.8% (n = 58) believed that antibiotics were useful in this context, while 30.1% (n = 63) were uncertain. Participants’ responses to all six items are presented in Table 3. The item on stopping antibiotics after symptom improvement and the two HIV/prophylaxis-specific items are context-dependent and are therefore interpreted cautiously rather than as definitive measures of knowledge.

For the two HIV/prophylaxis-specific items, 58.9% (n = 123) rejected the statement that PLWH need antibiotics more frequently than other individuals, whereas 10.5% (n = 22) agreed and 30.6% (n = 64) answered “do not know”. Similarly, 68.4% (n = 143) rejected the statement that preventive antibiotic use can avoid future illness, while 10.5% (n = 22) agreed and 21.1% (n = 44) were uncertain. These responses cannot distinguish inappropriate routine prevention from clinically indicated antimicrobial prophylaxis in selected patients.

The mean exploratory six-item antibiotic-related knowledge index was 3.63 ± 1.69 points, with a median of 4 points (interquartile range [IQR] 3–5; range 0–6). Index values differed across educational levels (Kruskal–Wallis H = 30.8, df = 4, *p* < 0.001) and were higher among participants from urban than rural areas (median 4 [IQR 3–5] vs. 3 [IQR 2–4]; Mann–Whitney *p* = 0.009). There was no clear evidence of an association between the six-item index and frequent self-reported antibiotic use (unadjusted OR 1.16 per point, 95% CI 0.88–1.54; *p* = 0.290). Sensitivity analyses gave the same overall conclusion after excluding the two context-dependent HIV/prophylaxis items (four-item index: OR 1.14, 95% CI 0.79–1.65; *p* = 0.475) and after additionally excluding the treatment-discontinuation item (three-item index: OR 0.99, 95% CI 0.65–1.52; *p* = 0.977).

### 2.6. Exploratory Adjusted Analyses of Frequent Self-Reported Antibiotic Use

An exploratory multivariable binary logistic regression analysis was performed for frequent self-reported antibiotic use (≥3 occasions during the preceding 12 months). Given the limited number of outcome events, a parsimonious model included two variables: at least one self-reported acute infection and suboptimal physician-estimated ART adherence (<95%). The complete-case analysis included 192 participants, of whom 22 met the outcome definition. Seventeen participants were excluded from this model alone because physician-estimated ART adherence was unavailable and/or the antibiotic-use response was non-evaluable.

In the primary adjusted model, self-reported acute infection was associated with frequent self-reported antibiotic use (adjusted odds ratio [aOR] 4.22, 95% CI 1.48–12.00; *p* = 0.007), as was suboptimal physician-estimated ART adherence (aOR 6.02, 95% CI 1.84–19.68; *p* = 0.003) (Table 4). Because these estimates were based on only 22 outcome events, they are interpreted as exploratory. Firth penalized-likelihood sensitivity analysis yielded similar estimates for acute infection (OR 3.90, 95% CI 1.42–10.73; *p* = 0.008) and suboptimal physician-estimated ART adherence (OR 5.88, 95% CI 1.83–18.87; *p* = 0.003). When the outcome comparison was restricted to no antibiotic use versus ≥3 occasions, the corresponding Firth estimates were OR 5.92 (95% CI 2.01–17.46; *p* = 0.001) and OR 6.67 (95% CI 1.65–26.97; *p* = 0.008), respectively. Separate Firth models adding age, CD4 ≤ 350 cells/mm^3^, or at least one recorded coinfection/comorbidity did not materially change the direction of the two primary associations. Full exploratory univariable and sensitivity analyses are provided in Supplementary File S3.

## 3. Discussion

This study provides an integrated description of self-reported acute infectious morbidity, usual healthcare-contact patterns, frequency of antibiotic use, and antibiotic-related knowledge among PLWH receiving routine care in a contemporary ART setting. The principal findings were the predominance of respiratory infections, the association of frequent self-reported antibiotic use with reported infectious morbidity and physician-estimated ART adherence in a sparse exploratory adjusted model, and the absence of clear evidence of an association with the exploratory knowledge index. The study did not assess antibiotic appropriateness, overprescribing, or guideline concordance.

The predominance of respiratory infections is consistent with evidence that lower respiratory tract infections and community-acquired pneumonia remain clinically relevant in the ART era. Lamas et al. showed that lower respiratory tract infection continued to occur among adults receiving ART and that higher CD4 counts and virological suppression were protective [5]. Sweet et al. likewise found broadly similar rates of acute respiratory infection encounters in people with and without HIV, but substantially more frequent infections among PLWH without viral suppression [13]. More recent longitudinal data indicate that community-acquired pneumonia is concentrated among individuals with poor immune recovery, whereas rates in those with preserved CD4 counts and sustained ART may approach those of the general population [6]. The absence of clear evidence for a relationship between acute infections and HIV-1 RNA in the present cohort may therefore reflect the predominance of controlled HIV infection and the limited number of participants with advanced immunosuppression.

Infection-related hospitalization for PLWH is also clinically important. Severe non-AIDS bacterial infections continue to be important causes of hospital admission among PLWH, particularly pneumonia, bloodstream infections, and urinary tract infections [8]. At the same time, the prognosis of common bacterial infections appears increasingly similar in well-controlled HIV infection. Bai et al. found that HIV status alone was not independently associated with in-hospital mortality among patients admitted with community-acquired pneumonia, whereas AIDS remained an important adverse prognostic factor [20]. The absence of a detectable difference in hospitalization according to the CD4 threshold used in our analysis should therefore be interpreted in the context of generally preserved immunity, the small number of hospitalization events, and substantial imprecision in the lower CD4 categories, rather than as evidence that immune status is irrelevant.

Family physicians were the most frequently selected usual healthcare contact for acute infections and the most frequently selected source of antibiotic prescription or recommendation. These findings place primary care and HIV specialist care within the same outpatient pathway but do not allow comparison of prescribing quality or provider superiority. Reviews of acute respiratory infection prescribing identify patient expectations, diagnostic uncertainty, workload, and time pressure as potential drivers of unnecessary treatment in primary care [21], and Romanian family physicians have described insufficient patient education and public awareness as contributors to antibiotic consumption [18]. Keintz et al. reported more guideline-concordant treatment and shorter antibiotic courses when PLWH with acute respiratory illness were managed by HIV clinicians rather than non-HIV clinicians [12]. In our setting, the practical implication is therefore a shared-care approach based on common prescribing principles, communication between family physicians and HIV clinicians, and concise patient information, rather than referral of routine acute illness to one provider group.

Usual healthcare-contact patterns also appeared to differ according to educational attainment. This pattern is plausible because health literacy may influence navigation of the healthcare system, confidence in primary care, and reliance on specialist services. Muflih et al. found that adequate health literacy was independently associated with better understanding of antibiotic use and antimicrobial resistance [22], while a multicountry survey in the WHO European Region identified physicians as the most trusted source of antibiotic information [17]. The association observed in our cohort should nevertheless be regarded as exploratory, because several education-by-provider categories were small and the pattern may also reflect continuity of care, perceived HIV-related vulnerability, or differential access to primary care.

The association between self-reported infectious morbidity and frequent self-reported antibiotic use is clinically plausible and also expected, because infections create opportunities for antibiotic treatment. Both variables were reported for the same 12-month period, so shared recall, healthcare utilization, and diagnostic labeling may have strengthened the observed relationship. The association with suboptimal physician-estimated ART adherence is clinically interesting but should likewise be interpreted cautiously. It is directionally consistent with previous evidence linking poorer ART adherence to opportunistic infection burden [23] and with cohort data showing more frequent respiratory encounters among PLWH without viral suppression [13]. The cross-sectional design cannot establish temporality, and the adherence measure may function as a broader care-vulnerability or engagement signal, reflecting clinical complexity or social vulnerability in addition to medication-taking behavior. In sensitivity Firth models, separate additional adjustment for age, CD4 category, or recorded coinfection/comorbidity did not materially change the direction of the associations, but residual confounding remains substantial. The very small <80% adherence subgroup contained no participants with frequent self-reported antibiotic use; therefore, the findings do not demonstrate a dose–response gradient and should be regarded as hypothesis-generating. The imprecision of the CD4 comparison is also consistent with the limited number of participants with advanced immunosuppression in this cohort [5,6].

The item-level responses identified uncertainty regarding antibiotic indications, particularly use for viral respiratory infections. Similar knowledge gaps have been reported in Romanian population studies and across the WHO European Region [15,16,17]. The six-item total, however, combines indications, resistance, treatment behavior, and HIV-specific beliefs and should not be regarded as a validated unidimensional knowledge scale. In addition, three items are context-dependent: clinically indicated prophylaxis may be appropriate for selected PLWH, and treatment duration may appropriately change after clinical reassessment. Because the wording administered to participants cannot be changed retrospectively, we retained the original items for transparency, treated the total as an exploratory index, emphasized item-level findings, and performed reduced-index sensitivity analyses. These analyses did not reveal clear evidence of an association with frequent self-reported antibiotic use. This cautious interpretation is also consistent with broader literature showing that antibiotic knowledge, beliefs, and behavior do not necessarily move in parallel [24,25,26].

The HIV/prophylaxis-specific responses require particular caution. Some participants agreed that PLWH need antibiotics more frequently or that preventive antibiotic use can avoid illness, but these statements do not distinguish nonspecific self-directed prevention from evidence-based prophylaxis for defined clinical or immunological indications. Current NIH guidance restricts antimicrobial prophylaxis to specific indications with criteria for initiation and discontinuation [27]. These items therefore identify areas requiring clearer patient-clinician communication rather than permitting a simple classification of all affirmative responses as misconceptions.

Higher exploratory knowledge-index values among participants with greater educational attainment and among urban residents are consistent with previous studies linking education, health literacy, and access to information with more accurate antibiotic knowledge [15,16,22]. However, there was no clear evidence of an association between the knowledge index and frequent self-reported antibiotic use. Thorpe et al. found that frequent users had greater health-literacy needs and were more inclined than non-users to request antibiotics for a hypothetical viral respiratory infection [28]; importantly, their frequent-use comparison was ≥3 uses versus no use rather than the 0–2 reference group used in our primary analysis. Our sensitivity analysis using the same 0 versus ≥3 comparison did not materially alter the main adjusted findings. These results support treating knowledge and antibiotic-use frequency as related but distinct constructs.

The WHO AWaRe framework provides practical guidance on antibiotic choice, dose, route, and duration for common infections [14], but implementation also requires communication and prescribing consistency across care settings. Systematic reviews indicate that combined patient-clinician education, point-of-care testing, electronic decision support, and clinician-facing interventions can improve prescribing, whereas patient education alone has less consistent effects on prescribing behavior [29,30]. These interventions are plausible directions for future shared-care stewardship research; their effectiveness was not tested in the present study.

A recently published population-based study by Valls-Carbó et al. linked pharmacy and microbiology data and reported markedly higher antibiotic consumption among people with HIV and pre-exposure prophylaxis (PrEP) users who had documented rectal sexually transmitted infection (STI) episodes than among general-population controls, with tetracyclines accounting for most of the difference; consumption among people with HIV and PrEP users without documented STIs was only modestly higher than in controls [31]. Our questionnaire did not collect episode-level indications, antibiotic classes, or systematic STI history, and therefore could not identify this source of exposure. This difference underscores the complementary nature of questionnaire-based and linked-record approaches and supports future prospective studies combining patient-reported pathways with pharmacy, microbiology, and medical-record data.

This study has several limitations. Its cross-sectional, single-center design limits causal inference and generalizability, and no formal a priori sample-size calculation was performed. Acute infections and antibiotic use were self-reported over the same preceding 12-month period, creating recall and classification bias and common healthcare-utilization effects. Reported infectious diagnoses were not independently verified or classified as clinical versus laboratory-confirmed. The antibiotic-use question did not collect episode-level indication, agent, dose, duration, route, prescribing setting, microbiological diagnosis, guideline concordance, or therapeutic versus prophylactic use; therefore, prescribing appropriateness and overprescribing cannot be estimated. Only one prescription/recommendation source was recorded per antibiotic user. The questionnaire was purpose-built, was not formally pilot-tested or psychometrically validated, and was administered face-to-face with the treating physician, so interviewer and social-desirability bias cannot be excluded. The exploratory knowledge index contains context-dependent items. Physician-estimated ART adherence was not based on a validated instrument and may reflect broader care engagement. Smoking, systematic STI history, antimicrobial prophylaxis, and prior bacterial infection history outside the preceding 12-month recall window were not systematically collected; vaccination information was collected in a separate module but was not incorporated into the present analysis. Although coinfections/comorbidities were recorded and explored, residual confounding by comorbidity burden and healthcare utilization remains possible. The small number of participants with frequent self-reported antibiotic use constrained adjusted analyses, produced wide confidence intervals, and left residual confounding by age, comorbidity, immune/virological status, healthcare utilization, and other unmeasured factors. Missing clinical data, including unavailable opportunistic-infection history in 46 participants, may have introduced additional bias; the original reason for historical-data unavailability could not be reconstructed reliably and these values were therefore retained as missing. Finally, the lack of an HIV-negative comparator precluded direct comparison with the general population.

## 4. Materials and Methods

### 4.1. Study Design and Setting

The “Prof. Dr. Matei Balș” National Institute of Infectious Diseases is a tertiary referral center specializing in infectious diseases and one of the major centers providing longitudinal HIV care in Romania. Approximately 3950 people living with HIV are actively monitored at the Institute, corresponding to about 21% of the 18,768 people living with HIV reported nationally in 2024 [19].

We conducted a single-center cross-sectional observational study based on a structured questionnaire administered to adult PLWH receiving care at the Institute between 15 October 2025, and 31 March 2026. Eligible participants were adults aged ≥18 years with confirmed HIV infection who were receiving routine care at the outpatient day-care unit and were able to understand and complete the questionnaire. Consecutive eligible patients were invited during the predefined enrollment period. No formal a priori sample-size calculation was performed. The manuscript was revised in accordance with STROBE recommendations for cross-sectional studies; a completed reporting checklist is provided as Supplementary File S4.

### 4.2. Questionnaire and Data Collection

The questionnaire comprised two complementary sections. The patient-reported section was completed during the routine day-care visit together with the treating infectious diseases physician, who routinely followed the patient and recorded the participant’s responses on the questionnaire form. Participants could request clarification during completion. The physician-completed section was filled in using clinical information available in the Institute’s electronic medical record. Each questionnaire was assigned a study code and contained no direct patient identifiers. After completion, data were entered into a de-identified electronic database; the study code was not retained as a linkage to the patient’s name or medical record.

Participants self-reported whether they had experienced respiratory, digestive, urinary, or other acute infections during the preceding 12 months and provided an approximate number of episodes within each category. They were also asked whether they had been diagnosed during the same period with selected specific infections, including influenza, COVID-19, acute pneumonia, acute otitis, acute sinusitis, acute gastroenteritis, and urinary tract infection. The questionnaire did not distinguish between clinically diagnosed, laboratory-confirmed, and self-perceived diagnoses. Participants were also asked about hospitalizations for acute infections. Independently of whether they had reported an acute infection during the preceding 12 months, all participants were asked whom they usually contact when experiencing an acute infection.

The questionnaire recorded the self-reported number of occasions on which antibiotics had been taken during the preceding 12 months using predefined categories (0, 1–2, 3–5, >5, or unable to recall). Participants reporting antibiotic use selected one source of prescription or recommendation from predefined response options. The instrument did not collect episode-level information on the antibiotic agent, indication, dose, duration, route, treatment setting, microbiological confirmation, or guideline concordance. It also did not separately identify inpatient therapy, opportunistic-infection prophylaxis, sexually transmitted infection treatment or prophylaxis, dental or surgical prophylaxis, topical therapy, or antibiotic use obtained outside the study institution. Consequently, the study assessed the frequency of self-reported antibiotic use and not prescribing appropriateness.

The physician-completed section included HIV-related clinical data obtained from the institutional electronic medical record, including the most recent available plasma HIV-1 RNA measurement and CD4 lymphocyte count, coinfections/comorbidities, history of opportunistic infections, current ART regimen, estimated treatment adherence, and treatment interruptions during the preceding 12 months. HIV-1 RNA was measured using the cobas^®^ HIV-1 quantitative assay on the cobas^®^ 6800 System. Results reported as “target not detected” were classified as undetectable. Any result with detectable HIV-1 RNA, including values below the lower limit of quantification (<20 copies/mL), was classified as detectable. Detectable results were recorded using the predefined questionnaire categories <1000 or >1000 copies/mL; the instrument did not specify a separate category for an exact value of 1000 copies/mL. ART adherence was estimated by the treating infectious diseases physician from the patient’s longitudinal treatment history available in the electronic medical record and categorized as ≥95%, 80–94%, <80%, or unknown. No validated adherence scale or prespecified recall interval was used; this was a pragmatic physician-estimated clinical measure.

The questionnaire was purpose-built for the study by physicians with expertise in epidemiology and infectious diseases, based on the study objectives and relevant literature regarding antibiotic use, acute infections, preventive behaviors, and HIV care. It was internally reviewed for content relevance, clarity, and comprehensibility. No formal pilot testing, patient-based content-validity assessment, or psychometric validation was performed before study initiation. The patient-reported section was administered face-to-face with the treating physician, and participants could request clarification; therefore, interviewer and social-desirability effects cannot be excluded. The original instrument was developed and administered in Romanian; no translation or back-translation was required for study administration. The complete questionnaire is provided as Supplementary Material in the original Romanian version (Supplementary File S1) and as an English translation prepared for transparency (Supplementary File S2). The broader questionnaire also included vaccination, COVID-19-related, and health-information modules that were analyzed separately and are not duplicated in the present study.

### 4.3. Ethical Considerations

The study protocol was approved by the Ethics Committee of the “Prof. Dr. Matei Balș” National Institute of Infectious Diseases prior to patient enrollment (approval number: C11346/08.10.2025). All participants provided written informed consent before inclusion. All study procedures were conducted in accordance with the ethical principles of the Declaration of Helsinki.

### 4.4. Statistical Analysis

Categorical variables were summarized using absolute frequencies and percentages, while continuous variables were expressed as mean ± standard deviation (SD) or median and range/interquartile range, as appropriate. The primary exploratory outcome was frequent self-reported antibiotic use, operationally defined a priori as antibiotic use on at least three occasions during the preceding 12 months. Participants reporting 0–2 occasions formed the reference group in the primary analysis. The ≥3 threshold was selected as a pragmatic marker of frequent use and was informed by recent survey research that defined frequent users as those reporting at least three antibiotic uses, although that study compared frequent users with non-users [28]. Participants who answered “do not know” were excluded from analyses involving this outcome. Wilson 95% confidence intervals were calculated for key prevalence estimates. A sensitivity analysis reproduced the literature comparison by restricting the reference group to participants reporting no antibiotic use (0 versus ≥3 occasions).

Comparisons between groups were performed using the chi-square test or Fisher’s exact test for categorical variables, depending on expected cell frequencies. Differences in antibiotic knowledge scores across educational categories were assessed using the Kruskal–Wallis test, while comparisons between two independent groups, including urban versus rural residence, were performed using the Mann–Whitney U test.

An exploratory antibiotic-related knowledge index ranging from 0 to 6 was constructed from the six questionnaire items. For each item, one response was assigned one point according to the prespecified scoring scheme, while the alternative response or “do not know” received zero points. Because the instrument was purpose-built and the items span several domains, the total was treated as an exploratory index rather than a validated unidimensional scale. Sensitivity analyses recalculated the index after excluding the two context-dependent HIV/prophylaxis items (four-item index) and after additionally excluding the treatment-discontinuation item (three-item index).

Candidate variables were not specified in a preregistered statistical analysis plan. The two variables retained in the primary adjusted model—occurrence of at least one self-reported acute infection and suboptimal physician-estimated ART adherence (<95%)—were selected on the basis of clinical relevance and review of the exploratory univariable findings, while limiting the number of parameters because only 22 participants met the outcome. The primary analysis used binary logistic regression with complete cases. Seventeen participants were excluded from this model alone because physician-estimated ART adherence was unavailable (n = 16) and/or the antibiotic-use response was non-evaluable (n = 2), with one participant meeting both criteria; participants were otherwise retained in descriptive and exploratory analyses whenever the relevant data were available. Because of sparse outcome events, Firth bias-reduced penalized-likelihood logistic regression using the same two covariates was performed as a sensitivity analysis. 95% confidence intervals were calculated for the Firth penalized estimates. Additional Firth models separately added age, CD4 ≤ 350 cells/mm^3^, and the presence of at least one recorded coinfection/comorbidity to assess residual confounding without fitting an overparameterized model. A further adherence sensitivity analysis compared the 80–94% category with ≥95% while excluding the very small <80% group. Univariable associations, missing-data patterns, and comparisons of participants included versus excluded from the complete-case model are reported in Supplementary File S3. Odds ratios (ORs) and 95% confidence intervals (95% CIs) were calculated. All subgroup and univariable analyses were considered exploratory. A two-sided *p*-value < 0.05 was used descriptively; no adjustment for multiple comparisons was applied, and *p*-values from the multiple exploratory tests should not be interpreted confirmatorily. Primary analyses were performed using Jamovi version 2.7.18.0 (The jamovi project, Sydney, Australia). Firth bias-reduced logistic regression sensitivity analyses were performed in R version 4.6.1 (R Foundation for Statistical Computing, Vienna, Austria).

## 5. Conclusions

In this exploratory study of PLWH receiving contemporary HIV care, frequent self-reported antibiotic use was associated with reported acute infections and suboptimal physician-estimated ART adherence, whereas there was no clear evidence of an association with the exploratory antibiotic-related knowledge index. These findings suggest that repeated antibiotic use in this population may reflect a combination of infectious morbidity and care-related factors and may not be explained by factual antibiotic knowledge alone. The involvement of both family physicians and HIV clinicians in the management of acute infections highlights the potential value of coordinated antimicrobial stewardship, including consistent prescribing principles, communication across care settings, and targeted patient counseling. Prospective studies linking individual infectious episodes to antibiotic indications, drug choice, treatment duration, microbiological findings, and guideline concordance are needed to identify where stewardship interventions could be most effective.

## Figures and Tables

**Table 1:** Sociodemographic and HIV-related characteristics of the study population.

Characteristic	Category	n (%)
**Age, years**	Mean ± SD	42.2 ± 12.1
	Median (range)	40 (20–77)
**Sex**	Male	155 (74.2)
	Female	54 (25.8)
**Area of residence**	Urban	161 (77.0)
	Rural	48 (23.0)
**Educational level**	No formal education	7 (3.3)
	Primary school	9 (4.3)
	Secondary school	33 (15.8)
	High school	96 (45.9)
	University education	63 (30.1)
	Data unavailable	1 (0.5)
**Occupational status**	Employed	88 (42.1)
	Retired	80 (38.3)
	Unemployed	15 (7.2)
	No occupation	21 (10.0)
	Student	4 (1.9)
	Data unavailable	1 (0.5)
**Duration since HIV diagnosis**	<1 year	19 (9.1)
	1–5 years	56 (26.8)
	6–10 years	39 (18.7)
	>10 years	95 (45.5)
**ART duration >10 years**		90 (43.1)
**HIV-1 RNA**	Undetectable	140/190 (73.7)
	Detectable, <1000 copies/mL	39/190 (20.5)
	>1000 copies/mL	11/190 (5.8)
	Data unavailable	19/209 (9.1)
**CD4 cell count**	<200 cells/mm^3^	14/194 (7.2)
	200–350 cells/mm^3^	18/194 (9.3)
	351–500 cells/mm^3^	33/194 (17.0)
	>500 cells/mm^3^	129/194 (66.5)
	Data unavailable	15/209 (7.2)
**Physician-estimated ART adherence**	≥95%	173/193 (89.6)
	80–94%	15/193 (7.8)
	<80%	5/193 (2.6)
	Data unavailable or not reliably estimated	16/209 (7.7)
**ART interruption during the preceding 12 months**	Yes	13/191 (6.8)
	No	178/191 (93.2)
	Data unavailable	18/209 (8.6)
**History of opportunistic infections**	Yes	11/163 (6.7)
	No	152/163 (93.3)
	Data unavailable	46/209 (22.0)

Data are presented as n (%) unless otherwise indicated. For clinical variables with missing data (HIV-1 RNA, CD4 cell count, physician-estimated ART adherence, ART interruption, and history of opportunistic infections), percentages for available categories were calculated using participants with available or evaluable data as the denominator, and cells are shown as n/N (%); unavailable data are reported relative to the total study population (N = 209). All other percentages use N = 209 unless otherwise specified. Duration since HIV diagnosis and ART duration were based on the patient-reported questionnaire items. ART, antiretroviral therapy; SD, standard deviation.

**Table 2:** Acute infections and antibiotic use during the preceding 12 months, and usual healthcare contact.

Domain	Variable/Category	n (%)
**Acute infection frequency**	At least one acute infection category	95 (45.5)
	At least two acute infection categories	24 (11.5)
**Acute infection categories**	Respiratory infections	60 (28.7)
	Digestive infections	21 (10.0)
	Urinary infection category	20 (9.6)
	Other acute infections	21 (10.0)
**Specific infectious diagnoses**	Urinary tract infection (specific diagnosis item)	17 (8.1)
	Influenza	16 (7.7)
	Acute gastroenteritis	16 (7.7)
	Acute sinusitis	15 (7.2)
	Acute pneumonia	11 (5.3)
	COVID-19	8 (3.8)
	Acute otitis	7 (3.3)
**Hospitalization for acute infections**	Any hospitalization (≥1)	20 (9.6)
	No hospitalization	189 (90.4)
	One hospitalization	15 (7.2)
	Two to three hospitalizations	3 (1.4)
	At least four hospitalizations	2 (1.0)
**Usual healthcare contact for an acute infection**	Family physician	115 (55.0)
	Infectious diseases physician	66 (31.6)
	Emergency department	14 (6.7)
	Another medical specialist	6 (2.9)
	Self-treatment	5 (2.4)
	Pharmacist	3 (1.4)
**Antibiotic-use occasions during the preceding 12 months**	None	99 (47.4)
	One to two occasions	86 (41.1)
	Three to five occasions	17 (8.1)
	More than five occasions	5 (2.4)
	Do not know/could not recall	2 (1.0)
**Frequent self-reported antibiotic use among evaluable participants**	≥3 occasions during preceding 12 months	22/207 (10.6)
Single selected source of antibiotic prescription or recommendation *	Family physician	41 (38.0)
	Another medical specialist	32 (29.6)
	Infectious diseases physician	25 (23.1)
	Self-medication	6 (5.6)
	Pharmacist recommendation	4 (3.7)

Data are presented as n (%) and were calculated using the total study population (N = 209), unless otherwise specified. Infection categories were not mutually exclusive because participants could report more than one category or diagnosis. Broad infection categories and predefined specific diagnoses were collected in separate questionnaire items and therefore were not expected to correspond exactly. “Any hospitalization (≥1)” is the aggregate of participants reporting exactly one, two to three, or at least four hospitalizations. Usual healthcare contact was reported by all 209 participants and referred to whom they generally contact when experiencing an acute infection, irrespective of whether an acute infection had been reported during the preceding 12 months. Frequent self-reported antibiotic use was calculated among the 207 participants with evaluable antibiotic-use responses. * Among the 108 participants reporting antibiotic use, one source of prescription or recommendation was selected from the predefined categories; the questionnaire did not capture different sources across multiple antibiotic-use occasions.

**Table 3:** Responses to antibiotic-related questionnaire items and prespecified scoring among study participants.

Statement	Scoring Response	1-Point Response, n (%)	Alternative Response, n (%)	Do Not Know, n (%)
**Antibiotics are effective only against bacterial infections**	True	107 (51.2)	32 (15.3)	70 (33.5)
**Antibiotics are useful for viral respiratory infections**	False	88 (42.1)	58 (27.8)	63 (30.1)
**Stopping antibiotic treatment when symptoms improve is acceptable**	False	151 (72.2)	35 (16.7)	23 (11.0)
**Frequent antibiotic use may lead to antimicrobial resistance**	True	146 (69.9)	21 (10.0)	42 (20.1)
**People living with HIV need antibiotics more frequently than other individuals**	False	123 (58.9)	22 (10.5)	64 (30.6)
**Preventive antibiotic use can help avoid future illness**	False	143 (68.4)	22 (10.5)	44 (21.1)

Data are presented as numbers and percentages of the total study population (N = 209). The “1-point response” column reflects the prespecified scoring scheme used for the exploratory six-item index. Because some items are context-dependent, this scoring scheme should not be interpreted as defining universally correct or incorrect responses or as a validated scale. “Do not know” responses were analyzed separately in this table but received zero points in the exploratory index. The statements are shown in a concise English rendering for presentation; the verbatim administered Romanian wording and the full English translation are provided in Supplementary Files S1 and S2.

**Table 4:** Exploratory multivariable logistic regression analysis of factors associated with frequent self-reported antibiotic use.

Variable	Adjusted OR	95% CI	*p*-Value
**At least one self-reported acute infection during the preceding 12 months**	4.22	1.48–12.00	0.007
**Suboptimal physician-estimated ART adherence (<95%)**	6.02	1.84–19.68	0.003

Frequent self-reported antibiotic use was operationally defined as antibiotic use on at least three occasions during the preceding 12 months. The reference categories were no self-reported acute infection and physician-estimated ART adherence ≥ 95%. The model included 192 participants with complete data, including 22 with frequent self-reported antibiotic use. Given the sparse number of outcome events and the exploratory, data-informed covariate selection, these estimates should not be interpreted as causal or definitive independent effects.

## Data Availability

The de-identified data underlying the findings of this study are available from the corresponding authors on reasonable request, subject to applicable ethical and institutional requirements. The full questionnaire is provided as Supplementary Files S1 and S2.

## Supplementary Materials

The following supporting information can be downloaded at: https://www.mdpi.com/article/10.3390/antibiotics15100979/s1, File S1: Questionnaire (original Romanian version); File S2: Questionnaire (English translation); File S3: Additional exploratory and sensitivity analyses; File S4: STROBE reporting checklist for cross-sectional studies.
