# Supplementary material for: Acute Infections, Antibiotic Use and Antibiotic-Related Knowledge Among People Living with HIV: A Cross-Sectional Questionnaire-Based Study
Source: Antibiotics (Basel). 2026 Oct 3;15(10):979. doi: 10.3390/antibiotics15100979 (PMC13700600; doi:10.3390/antibiotics15100979)
Supplement: Supplementary file 1 [file antibiotics-15-00979-s001.zip › Supplementary.S1_Questionnaire.Romanian.pdf]

No [_____________]

Chestionar privind impactul bolilor infecțioase acute

Partea 1 - Se completează de către pacient

1. Sex: ☐ Masculin / ☐ Feminin

2. Vârsta: [____] ani

3. Mediu de domiciliu: ☐ Urban / ☐ Rural

4. Nivel de educație, ultima școală absolvită:
☐ Fără școala / ☐ Școala primară / ☐ Școala gimnazială / ☐ Liceu / ☐ Facultate, master

5. Ocupație:
☐ Angajat / ☐ Șomer / ☐ Pensionar / ☐ Student / ☐ Fără ocupație

6. Durata de timp de la diagnosticul infecției HIV:
☐ <1 an / ☐ 1–5 ani / ☐ 6–10 ani / ☐ >10 ani

7. Durata de timp de la inițierea terapiei HIV:
☐ <1 an / ☐ 1–5 ani / ☐ 6–10 ani / ☐ >10 ani

8. În ultimul an, ați avut infecții acute: (bifați toate variantele aplicabile)

- respiratorii? ☐ DA / ☐ NU; dacă DA, numărul aproximativ [____]
- digestive? ☐ DA / ☐ NU; dacă DA, numărul aproximativ [____]
- urinare? ☐ DA / ☐ NU; dacă DA, numărul aproximativ [____]
- alte infecții? ☐ DA / ☐ NU; dacă DA, precizați ce fel de infecți și numărul aproximativ [____]

9. În ultimul an ați fost diagnosticat cu:

- Gripă ☐ DA / ☐ NU
- COVID-19 ☐ DA / ☐ NU
- Pneumonie acută ☐ DA / ☐ NU
- Otită acută ☐ DA / ☐ NU
- Sinuzită acută ☐ DA / ☐ NU
- Gastro-enterocolită acută ☐ DA / ☐ NU
- Infecție de tract urinar ☐ DA / ☐ NU

10. În ultimul an, câte spitalizări pentru infecții acute ați avut?

☐ 0 / ☐ 1 / ☐ 2–3 / ☐ ≥4

11. Când aveți o infecție acută, la cine apelați de obicei?

☐ Medicul de familie

☐ Medicul de boli infecțioase

☐ Urgențe (CPU/UPU)

☐ Alt medic specialist

☐ Farmacie, solicit recomandări de tratament

☐ Mă tratez singur

12. În ultimul an, de câte ori ați luat antibiotice?

☐ 0 / ☐ 1–2 / ☐ 3–5 / ☐ >5 / ☐ Nu știu

13. Antibioticele v-au fost prescrise de:

☐ Medicul de boli infecțioase

☐ Medicul de familie

☐ Alt medic specialist

☐ Farmacist

☐ Auto-medicație

14. Cum considerați următoarele afirmații?

**Afirmație**	**Adevărat**	**Fals**	**Nu știu**
Antibioticele tratează doar infecțiile bacteriene	☐	☐	☐
Antibioticele sunt utile în virozele respiratorii	☐	☐	☐
Oprirea antibioticelor când mă simt mai bine este în regulă	☐	☐	☐
Utilizarea frecventă a antibioticelor poate duce la rezistență	☐	☐	☐
Persoanele cu HIV trebuie să ia antibiotice mai des decât alții	☐	☐	☐
Dacă iau antibiotic preventiv, evit îmbolnăvirea	☐	☐	☐

15. În ultimii 3 ani ați făcut unul din următoarele vaccinuri...?

- vaccin gripal ☐ Da / ☐ Nu
- vaccin COVID-19 ☐ Da / ☐ Nu
- vaccin pneumococic ☐ Da / ☐ Nu
- vaccin meningococic ☐ Da / ☐ Nu
- vaccin hepatită B ☐ Da / ☐ Nu

- vaccin hepatită A ☐ Da / ☐ Nu
- vaccin varicelă ☐ Da / ☐ Nu
- vaccin HPV ☐ Da / ☐ Nu
- alt vaccin ☐ Da / ☐ Nu, Dacă DA, ce vaccin [________________________________]
  ☐ Nu am făcut niciun vaccin

16. Dacă NU v-ați vaccinat, principalele motive sunt (bifați toate variantele posibile):

☐ Teamă de reacții adverse
☐ Consider că nu e necesar
☐ Lipsă de recomandare din partea medicului
☐ Lipsă de timp
☐ Acces dificil
☐ Nu am încredere în vaccinuri
☐ Alt motiv: [____]

17. Cum evaluați următoarele afirmații?

**Afirmație**	**Acord**	**Neutru**	**Dezacord**
Persoanele cu HIV sunt mai vulnerabile la infecții	☐	☐	☐
Vaccinurile ajută la prevenirea spitalizărilor	☐	☐	☐
Informarea despre infecții îmi reduce anxietatea	☐	☐	☐
Recomandările medicului infecționist sunt suficiente pentru protecția mea	☐	☐	☐

18. Sunteți de acord sau nu cu următoarele afirmații?

**Afirmație**	**De acord**	**În dezacord**	**Nu știu**
Antiretroviralele mă protejează complet împotriva altor infecții	☐	☐	☐
Dacă am HIV, antibioticele sunt mai puțin eficiente	☐	☐	☐
Vaccinurile pot reactiva HIV	☐	☐	☐
E mai bine să tratez infecțiile când apar decât să mă vaccinez	☐	☐	☐

20. De unde vă informați cel mai des despre sănătate?

☐ Medicul de boli infecțioase
☐ Medicul de familie

☐ Alt medic specialist
☐ Internet / Social media
☐ Familie / Prieteni
☐ Altele: [____]

21. V-ați dori ca medicul de boli infecțioase să vă ofere mai multe informații despre prevenirea infecțiilor acute?
☐ Da / ☐ Nu / ☐ Nu sunt sigur

22. În prezent în spații aglomerate sau în mijloacele de transport purtați mască de protecție?
☐ Da / ☐ Nu

23. În perioada pandemiei COVID-19, ați evitat să veniți la spital pentru control?
☐ Da / ☐ Nu / ☐ Nu îmi amintesc

24. Ați avut dificultăți în a obține medicamentele antiretrovirale în timpul pandemiei de COVID-19?
☐ Da / ☐ Nu / ☐ Nu se aplică, nu luam tratament antiretroviral în acea perioadă

25. Cum apreciați accesul la servicii medicale în pandemie comparativ cu perioada actuală?
☐ Mult mai dificil / ☐ Puțin mai dificil / ☐ La fel / ☐ Mai ușor

26. Dacă în timpul pandemiei COVID-19 ați avut nevoie de îngrijiri urgente, ați reușit să le obțineți rapid?
☐ Da / ☐ Nu / ☐ Nu am avut nevoie

27. În timpul pandemiei, ați avut o infecție COVID-19 confirmată?
☐ Da, o singură dată / ☐ Da, de mai multe ori / ☐ Nu / ☐ Nu știu
28. Dacă DA: a fost necesară spitalizarea?
☐ Da ☐ Nu ☐ Nu se aplică

29. Pandemia v-a afectat starea generală de sănătate?
☐ Da, semnificativ / ☐ Da, într-o anumită măsură / ☐ Nu prea / ☐ Deloc

30. Pandemia a afectat relația cu medicii și comunicarea privind sănătatea?
☐ Da, negativ / ☐ Nu, a rămas la fel / ☐ Da, pozitiv (mai mult suport)

No [_____________]

Partea 2 - Se completează de către medic

1. Durata de timp de la diagnosticul infecției HIV:

☐ <1 an / ☐ 1–5 ani / ☐ 6–10 ani / ☐ >10 ani

2. Durata de timp de la inițierea terapiei HIV:

☐ <1 an / ☐ 1–5 ani / ☐ 6–10 ani / ☐ >10 ani

3. Ultima valoarea a încărcăturii virale (HIV-RNA):

☐ Nedetectabil / ☐ Detectabil < 1000 copii/mL / ☐ Detectabil >1000 copii/mL

4. Ultima valoare CD4:

☐ <200 ☐ 200-350 ☐ 351-500 ☐ >500

5. Co-infecții / comorbidități relevante: (bifați toate aplicabile)

☐ VHB

☐ VHC

☐ TBC

☐ Boli cardiovasculare

☐ Obezitate

☐ Diabet

☐ BPOC

☐ Neoplazii

☐ Altele: [____]

6. Istoric de infecții oportuniste: ☐ Da ☐ Nu

7. Regim ART actual: [____]

8. Aderență estimată de medic la ART:

☐ ≥95% (foarte bună) / ☐ 80–94% (parțială) / ☐ <80% (slabă) / ☐ Necunoscut

9. Întreruperi tratament ART în ultimul an:

☐ Da / ☐ Nu

10. Detalii relevante pentru interpretarea răspunsurilor pacientului: …
