# Supplementary material for: Acute Infections, Antibiotic Use and Antibiotic-Related Knowledge Among People Living with HIV: A Cross-Sectional Questionnaire-Based Study
Source: Antibiotics (Basel). 2026 Oct 3;15(10):979. doi: 10.3390/antibiotics15100979 (PMC13700600; doi:10.3390/antibiotics15100979)
Supplement: Supplementary file 1 [file antibiotics-15-00979-s001.zip › Supplementary.S2_Questionnaire_EnglishTranslation.pdf]

Supplementary File S2

Questionnaire on the Impact of Acute Infectious Diseases – English Translation

Part 1 – Completed by the patient

1. Sex: ☐ Male ☐ Female

2. Age: [____] years

3. Area of residence: ☐ Urban ☐ Rural

4. Educational level, highest school completed: ☐ No formal education ☐ Primary school ☐ Secondary school ☐ High school ☐ University/Master's degree

5. Occupation: ☐ Employed ☐ Unemployed ☐ Retired ☐ Student ☐ No occupation

6. Time since HIV diagnosis: ☐ <1 year ☐ 1–5 years ☐ 6–10 years ☐ >10 years

7. Time since initiation of HIV therapy: ☐ <1 year ☐ 1–5 years ☐ 6–10 years ☐ >10 years

8. During the preceding year, did you have acute infections? (check all that apply)

• Respiratory? ☐ YES / ☐ NO; if YES, approximate number [____]

• Digestive? ☐ YES / ☐ NO; if YES, approximate number [____]

• Urinary? ☐ YES / ☐ NO; if YES, approximate number [____]

• Other infections? ☐ YES / ☐ NO; if YES, specify the type of infection(s) and approximate number [______________________________]

9. During the preceding year, were you diagnosed with:

• Influenza ☐ YES / ☐ NO

• COVID-19 ☐ YES / ☐ NO

• Acute pneumonia ☐ YES / ☐ NO

• Acute otitis ☐ YES / ☐ NO

• Acute sinusitis ☐ YES / ☐ NO

• Acute gastroenteritis ☐ YES / ☐ NO

• Urinary tract infection ☐ YES / ☐ NO

10. During the preceding year, how many hospitalizations for acute infections did you have? ☐ 0 ☐ 1 ☐ 2–3 ☐ ≥4

11. When you have an acute infection, whom do you usually contact?

☐ Family physician

☐ Infectious diseases physician

☐ Emergency department (CPU/UPU)

☐ Another medical specialist

☐ Pharmacy – I request treatment recommendations

☐ I treat myself

12. During the preceding year, how many times did you take antibiotics? ☐ 0 ☐ 1–2 ☐ 3–5 ☐ >5 ☐ I do not know

13. The antibiotics were prescribed by:

☐ Infectious diseases physician

☐ Family physician

☐ Another medical specialist

☐ Pharmacist

☐ Self-medication

14. How do you consider the following statements?

Statement	True	False	Do not know
Antibiotics treat only bacterial infections	☐	☐	☐
Antibiotics are useful for viral respiratory infections	☐	☐	☐
Stopping antibiotics when I feel better is acceptable	☐	☐	☐
Frequent use of antibiotics can lead to resistance	☐	☐	☐
People with HIV need to take antibiotics more often than other people	☐	☐	☐
If I take antibiotics preventively, I avoid becoming ill	☐	☐	☐

15. During the past 3 years, have you received any of the following vaccines?

• Influenza vaccine ☐ Yes / ☐ No

• COVID-19 vaccine ☐ Yes / ☐ No

• Pneumococcal vaccine ☐ Yes / ☐ No

• Meningococcal vaccine ☐ Yes / ☐ No

• Hepatitis B vaccine ☐ Yes / ☐ No

• Hepatitis A vaccine ☐ Yes / ☐ No

• Varicella vaccine ☐ Yes / ☐ No

• HPV vaccine ☐ Yes / ☐ No

• Other vaccine ☐ Yes / ☐ No; if YES, which vaccine [____________________________]

☐ I have not received any vaccine

16. If you have NOT been vaccinated, what are the main reasons? (check all that apply)

☐ Fear of adverse reactions

☐ I do not consider it necessary

☐ Lack of recommendation from the physician

☐ Lack of time

☐ Difficult access

☐ I do not trust vaccines

☐ Other reason: [______________________________]

17. How do you evaluate the following statements?

Statement	Agree	Neutral	Disagree
People with HIV are more vulnerable to infections	☐	☐	☐
Vaccines help prevent hospitalizations	☐	☐	☐
Information about infections reduces my anxiety	☐	☐	☐
Recommendations from my infectious diseases physician are sufficient for my protection	☐	☐	☐

18. Do you agree or disagree with the following statements?

Statement	Agree	Disagree	Do not know
Antiretroviral therapy completely protects me against other infections	☐	☐	☐
If I have HIV, antibiotics are less effective	☐	☐	☐
Vaccines can reactivate HIV	☐	☐	☐
It is better to treat infections when they occur than to be vaccinated	☐	☐	☐

20. Where do you most often obtain health information?

☐ Infectious diseases physician

☐ Family physician

☐ Another medical specialist

☐ Internet / Social media

☐ Family / Friends

☐ Other: [______________________________]

21. Would you like your infectious diseases physician to provide you with more information about preventing acute infections? ☐ Yes ☐ No ☐ Not sure

22. At present, do you wear a protective mask in crowded spaces or on public transport? ☐ Yes ☐ No

23. During the COVID-19 pandemic, did you avoid coming to the hospital for follow-up? ☐ Yes ☐ No ☐ I do not remember

24. Did you have difficulty obtaining antiretroviral medication during the COVID-19 pandemic? ☐ Yes ☐ No ☐ Not applicable – I was not taking antiretroviral treatment at that time

25. How do you rate access to medical services during the pandemic compared with the current period? ☐ Much more difficult ☐ Slightly more difficult ☐ The same ☐ Easier

26. If you needed urgent medical care during the pandemic, were you able to obtain it promptly? ☐ Yes ☐ No ☐ I did not need urgent care

27. During the pandemic, did you have confirmed COVID-19 infection? ☐ Yes, once ☐ Yes, more than once ☐ No ☐ I do not know

28. If YES, was hospitalization required? ☐ Yes ☐ No ☐ Not applicable

29. Did the pandemic affect your general health? ☐ Yes, significantly ☐ Yes, to some extent ☐ Not much ☐ Not at all

30. Did the pandemic affect your relationship with physicians and communication regarding health? ☐ Yes, negatively ☐ No, it remained the same ☐ Yes, positively (more support)

Note on numbering: The original questionnaire did not contain an item numbered 19; the original numbering has been preserved in this translation.

Part 2 – Completed by the physician

1. Time since HIV diagnosis: ☐ <1 year ☐ 1–5 years ☐ 6–10 years ☐ >10 years

2. Time since initiation of HIV therapy: ☐ <1 year ☐ 1–5 years ☐ 6–10 years ☐ >10 years

3. Most recent HIV viral load (HIV-RNA): ☐ Undetectable ☐ Detectable <1000 copies/mL ☐ Detectable >1000 copies/mL

4. Most recent CD4 value: ☐ <200 ☐ 200–350 ☐ 351–500 ☐ >500

5. Relevant coinfections / comorbidities (check all that apply):

☐ HBV

☐ HCV

☐ Tuberculosis

☐ Cardiovascular disease

☐ Obesity

☐ Diabetes

☐ COPD

☐ Neoplasia

☐ Other: [______________________________]

6. History of opportunistic infections: ☐ Yes ☐ No

7. Current ART regimen: [____]

8. ART adherence estimated by the physician: ☐ ≥95% (very good) ☐ 80–94% (partial) ☐ <80% (poor) ☐ Unknown

9. ART treatment interruptions during the preceding year: ☐ Yes ☐ No

10. Details relevant to interpretation of the patient's responses:
[____]
