# Supplementary material for: Acute Infections, Antibiotic Use and Antibiotic-Related Knowledge Among People Living with HIV: A Cross-Sectional Questionnaire-Based Study
Source: Antibiotics (Basel). 2026 Oct 3;15(10):979. doi: 10.3390/antibiotics15100979 (PMC13700600; doi:10.3390/antibiotics15100979)
Supplement: Supplementary file 1 [file antibiotics-15-00979-s001.zip › Supplementary.S3_AdditionalAnalysesR2.pdf]

Supplementary File S3. Additional Exploratory and Sensitivity Analyses

Manuscript: Acute Infections, Antibiotic Use and Antibiotic-Related Knowledge among People Living with HIV

Table S3.1. Key prevalence estimates with 95% confidence intervals

Outcome	n/N	%	95% CI
At least one self-reported acute infection category	95/209	45.5	38.8–52.2
Self-reported respiratory infection	60/209	28.7	23.0–35.2
Hospitalization for an acute infection	20/209	9.6	6.3–14.3
Frequent self-reported antibiotic use (≥3 occasions)	22/207	10.6	7.1–15.6

Table S3.2. Exploratory univariable associations with frequent self-reported antibiotic use

Variable	N	Outcome/ exposed	Outcome/ reference	OR	95% CI	p
Age, per year	207	—	—	0.96	0.92–1.00	0.075
Female sex	207	3/54	19/153	0.41	0.12–1.46	0.204
Urban residence	207	18/159	4/48	1.40	0.45–4.37	0.790
High school/university education	206	14/157	8/49	0.50	0.20–1.28	0.183
HIV diagnosis >10 years	207	14/95	8/112	2.25	0.90–5.62	0.112
ART duration >10 years	207	11/90	11/117	1.34	0.55–3.25	0.650
At least one self-reported acute infection	207	16/95	6/112	3.58	1.34–9.56	0.011
Any hospitalization for acute infection	207	6/20	16/187	4.58	1.55–13.56	0.010
Detectable HIV-1 RNA	189	6/50	16/139	1.05	0.39–2.85	1.000
CD4 ≤350 cells/mm³	193	7/32	15/161	2.73	1.01–7.35	0.062
Any recorded coinfection/comorbidity	192	16/93	5/99	3.91	1.37–11.15	0.010
History of opportunistic infection	162	3/11	16/151	3.16	0.76–13.15	0.123
Suboptimal physician-estimated ART adherence (<95%)	192	6/19	16/173	4.53	1.51–13.55	0.011
ART interruption during preceding 12 months	190	4/13	18/177	3.93	1.10–14.05	0.048
Exploratory six-item knowledge index, per point	207	—	—	1.16	0.88–1.54	0.290

Note: For binary variables, "Outcome/exposed" and "Outcome/reference" give the number with frequent self-reported antibiotic use divided by the number in each comparison category. Fisher exact p-values are shown for binary variables. For continuous variables, p-values are from univariable logistic regression.

Table S3.3. Sensitivity analyses of the adjusted model

Analysis	Variable	OR/aOR	95% CI	p	N (events)
Primary logistic model (0–2 vs ≥3)	Acute infection	4.22	1.48–12.00	0.007	192 (22)
Primary logistic model (0–2 vs ≥3)	ART adherence <95%	6.02	1.84–19.68	0.003	192 (22)
Firth model (0–2 vs ≥3)	Acute infection	3.90	1.42–10.73	0.008	192 (22)
Firth model (0–2 vs ≥3)	ART adherence <95%	5.88	1.83–18.87	0.003	192 (22)
Firth outcome sensitivity (0 vs ≥3)	Acute infection	5.92	2.01–17.46	0.001	115 (22)
Firth outcome sensitivity (0 vs ≥3)	ART adherence <95%	6.67	1.65–26.97	0.008	115 (22)
Firth + age	Acute infection	3.90	1.42–10.67	0.008	192 (22)
Firth + age	ART adherence <95%	5.13	1.59–16.54	0.006	192 (22)
Firth + CD4 ≤350	Acute infection	3.76	1.36–10.38	0.010	191 (22)
Firth + CD4 ≤350	ART adherence <95%	4.87	1.46–16.24	0.010	191 (22)

Analysis	Variable	OR/aOR	95% CI	p	N (events)
Firth + coinfection/comorbidity	Acute infection	3.72	1.30–10.69	0.015	190 (21)
Firth + coinfection/comorbidity	ART adherence <95%	4.50	1.30–15.57	0.018	190 (21)
Adherence sensitivity (exclude <80%)	Acute infection	3.72	1.33–10.39	0.012	187 (22)
Adherence sensitivity (exclude <80%)	ART adherence 80–94% vs ≥95%	8.85	2.52–31.12	<0.001	187 (22)

Table S3.4. Sensitivity analyses of the exploratory antibiotic-related knowledge index

Index	Items retained	OR per point	95% CI	p
Six-item index	All six items	1.16	0.88–1.54	0.290
Four-item index	Excludes HIV-frequency and preventive-antibiotic items	1.14	0.79–1.65	0.475
Three-item index	Additionally excludes treatment-discontinuation item	0.99	0.65–1.52	0.977

Table S3.5. Missing or unavailable data for selected variables

Variable	Unavailable, n/N (%)
Educational level	1/209 (0.5%)
HIV-1 RNA	19/209 (9.1%)
CD4 count	15/209 (7.2%)
Coinfections/comorbidities	16/209 (7.7%)
History of opportunistic infections	46/209 (22.0%)
Current ART regimen	16/209 (7.7%)
Physician-estimated ART adherence	16/209 (7.7%)
ART interruption	18/209 (8.6%)
Antibiotic-use outcome	2/209 (1.0%)

Table S3.6. Comparison of participants included versus excluded from the primary complete-case adjusted model

Characteristic	Included (N=192)	Excluded (N=17)	p
Age, median (IQR), years	40 (34–50)	38 (34–47)	0.728
Female sex	52/192 (27.1%)	2/17 (11.8%)	0.249
Urban residence	147/192 (76.6%)	14/17 (82.4%)	0.768
At least one acute infection	90/192 (46.9%)	5/17 (29.4%)	0.208
Any hospitalization for acute infection	19/192 (9.9%)	1/17 (5.9%)	1.000
Knowledge index, median (IQR)	4 (3–5)	3 (2–5)	0.301
CD4 ≤350 cells/mm³, among available	31/191 (16.2%)	1/3 (33.3%)	0.420
Detectable HIV-1 RNA, among available	48/187 (25.7%)	2/3 (66.7%)	0.170
Any coinfection/comorbidity, among available	93/190 (48.9%)	1/3 (33.3%)	1.000

Note: The 17 participants excluded from the primary complete-case model were omitted from that model only because physician-estimated ART adherence was unavailable (n=16) and/or the antibiotic-use response was non-evaluable (n=2), with one participant meeting both criteria.

Table S3.7. Additional descriptive clinical information

Clinical characteristic	n/N (%)
At least one recorded coinfection/comorbidity	94/193 (48.7%)
Cardiovascular disease	20/193 (10.4%)
Hepatitis B virus infection	19/193 (9.8%)
Hepatitis C virus infection	14/193 (7.3%)
Diabetes	12/193 (6.2%)
Obesity	11/193 (5.7%)
Tuberculosis	10/193 (5.2%)

Clinical characteristic	n/N (%)
**Chronic obstructive pulmonary disease**	**8/193 (4.1%)**
**Neoplasia**	**3/193 (1.6%)**
**Bictegravir/emtricitabine/tenofovir alafenamide**	**72/193 (37.3%)**
**Dolutegravir/lamivudine**	**41/193 (21.2%)**
**Doravirine/lamivudine/tenofovir disoproxil**	**36/193 (18.7%)**
**Dolutegravir/abacavir/lamivudine**	**17/193 (8.8%)**
**Other or unspecified ART regimen**	**27/193 (14.0%)**

Note: Coinfection/comorbidity categories are not mutually exclusive. The denominator is 193 participants with available information for the respective physician-completed fields.
