# Supplementary material for: Acute Infections, Antibiotic Use and Antibiotic-Related Knowledge Among People Living with HIV: A Cross-Sectional Questionnaire-Based Study
Source: Antibiotics (Basel). 2026 Oct 3;15(10):979. doi: 10.3390/antibiotics15100979 (PMC13700600; doi:10.3390/antibiotics15100979)
Supplement: Supplementary file 1 [file antibiotics-15-00979-s001.zip › Supplementary.S4_STROBE.pdf]

Supplementary File S4. STROBE Reporting Checklist – Cross-Sectional Study

Manuscript: "Acute Infections, Antibiotic Use and Antibiotic-Related Knowledge among People Living with HIV: A Cross-Sectional Questionnaire-Based Study"

Item	Topic	Reporting requirement (paraphrased)	Location in revised manuscript
1a	Title/abstract	Identify the observational design.	Title; Abstract—Methods
1b	Title/abstract	Give a balanced abstract of methods and findings.	Abstract
2	Background	Explain scientific rationale.	Introduction
3	Objectives	State study objectives/hypotheses.	End of Introduction
4	Study design	Present key design features early.	Materials and Methods—4.1 Study Design and Setting
5	Setting	Describe center, location, dates, and recruitment period.	Materials and Methods—4.1 Study Design and Setting
6a	Participants	State eligibility and selection method.	Materials and Methods—4.1 Study Design and Setting
7	Variables	Define outcomes, covariates, and clinical categories.	Materials and Methods—4.2 Questionnaire and Data Collection; 4.4 Statistical Analysis
8	Data sources/measurement	Describe data sources and assessment methods.	Materials and Methods—4.2 Questionnaire and Data Collection
9	Bias	Describe potential bias and mitigation/limitations.	Materials and Methods—4.2 Questionnaire and Data Collection; Discussion, limitations paragraph
10	Study size	Explain how sample size was reached.	Materials and Methods—4.1 Study Design and Setting
11	Quantitative variables	Explain categorization/handling of quantitative variables.	Materials and Methods—4.4 Statistical Analysis; Supplementary File S3
12a	Statistical methods	Describe analyses and control of confounding.	Materials and Methods—4.4 Statistical Analysis
12b	Statistical methods	Describe subgroup/interaction analyses.	Materials and Methods—4.4 Statistical Analysis; Results; Supplementary File S3
12c	Statistical methods	Explain handling of missing data.	Materials and Methods—4.4 Statistical Analysis; Results; Supplementary File S3
12d	Statistical methods	Account for sampling strategy where relevant.	Not applicable: consecutive recruitment without complex sampling
12e	Statistical methods	Describe sensitivity analyses.	Materials and Methods—4.4 Statistical Analysis; Results; Supplementary File S3
13a	Participants	Report numbers at each stage.	Results—Study Population Characteristics
13b	Participants	Give reasons for non-participation.	Results—18/227 declined participation
13c	Participants	Consider a flow diagram.	Participant flow reported numerically; no separate diagram used
14a	Descriptive data	Report participant characteristics and potential confounders.	Table 1; Results—Study Population Characteristics
14b	Descriptive data	Report missing data by variable.	Results; Supplementary File S3—missing-data table
15	Outcome data	Report outcome frequencies/summary measures.	Results—acute infections, hospitalization, antibiotic use, knowledge
16a	Main results	Give crude/adjusted estimates with precision and adjustment rationale.	Results—Exploratory Adjusted Analyses; Table 4; Supplementary File S3
16b	Main results	Report category boundaries used in analysis.	Statistical Analysis; Results; table notes
16c	Main results	Translate relative estimates into absolute risk if relevant.	Not applicable for the exploratory cross-sectional logistic model
17	Other analyses	Report subgroup and sensitivity analyses.	Results; Supplementary File S3
18	Key results	Summarize findings in relation to objectives.	Discussion, opening paragraph; Conclusions
19	Limitations	Discuss bias, imprecision, and study limitations.	Discussion, limitations paragraph
20	Interpretation	Provide cautious interpretation considering limitations and multiplicity.	Discussion; Conclusions
21	Generalisability	Discuss external validity.	Discussion, limitations paragraph (single-center design, no comparator)
22	Funding	Report study/publication funding and role where applicable.	Acknowledgments
